# Futuristic biosensors for cardiac health care: an artificial intelligence approach

**DOI:** 10.1007/s13205-018-1368-y

**Published:** 2018-08-03

**Authors:** Rajat Vashistha, Arun Kumar Dangi, Ashwani Kumar, Deepak Chhabra, Pratyoosh Shukla

**Affiliations:** 1 Optimization and Mechatronics Laboratory, Department of Mechanical Engineering, University Institute of Engineering and Technology, Maharshi Dayanand University, Rohtak, Haryana India; 2 Enzyme Technology and Protein Bioinformatics Laboratory, Department of Microbiology, Maharshi, Dayanand University, Rohtak, Haryana 124001 India

**Keywords:** Biosensors, Artificial intelligence, The point of care diagnostics, Big data, Internet of things

## Abstract

Biosensor-based devices are pioneering in the modern biomedical applications and will be the future of cardiac health care. The coupling of artificial intelligence (AI) for cardiac monitoring-based biosensors for the point of care (POC) diagnostics is prominently reviewed here. This review deciphers the most significant machine-learning algorithms for the futuristic biosensors along with the internet of things, computational techniques and microchip-based essential cardiac biomarkers for real-time health monitoring and improving patient compliance. The present review also discusses the recently developed cardiac biosensors along with technical strategies involved in their mechanism of working and their applications in healthcare. Additionally, it provides a key for the ontogeny of an effective and supportive hierarchical protocol for clinical decision-making about personalized medicine through combinatory information analysis, and integrated multidisciplinary AI approaches.

## Introduction

Cardiovascular diseases (CVDs) and stroke are at the top causing death globally. World Health Organization (WHO), approximate death of 17.7 million people due to CVDs in 2015 which represented 31% of all global deaths (WHO 2017). Among these deaths, approximately 7.4 million were due to coronary heart disease, and 6.7 million were due to stroke (Gorgieva et al. 2018). Early and quick diagnosis is crucial for successful prognosis of CVD and stroke. In this regard, many cardiac-specific biomarkers such as myoglobin, B-type natriuretic peptide (BNP), cardiac troponin I (cTnI), C-reactive protein (CRP), and interleukins, interferons are identified which are detected using optical (colorimetric, fluorescence, luminescence, surface plasma resonance (SPR) and fiber optics/bio-optrode), acoustic (CMOS Si chips), electrochemical (potentiometric, amperometric and impedimetric transducers), and magnetic-based biosensors (Qureshi et al. 2012). Although significant advances in biosensors generations have been achieved, these face some serious limitations. Most of the developed biosensors follow a classical approach where tests are carried out in central laboratories that required several hours or days for final results. Further, for CVD diagnosis patients should meet at least two of three conditions: elevation of blood biomarker levels, characteristic chest pain and diagnostic electrocardiogram (ECG) alterations. But half of the CVD patients even admitted to emergency departments show normal ECG pattern which makes CVD diagnosis more difficult (Herring and Paterson 2006). Thus, there is the vital demand for more sensitive, reliable, cost-effective diagnostic platform which can also help in the real-time detection and monitoring of the health of CVD patients.

**Fig. 1:**
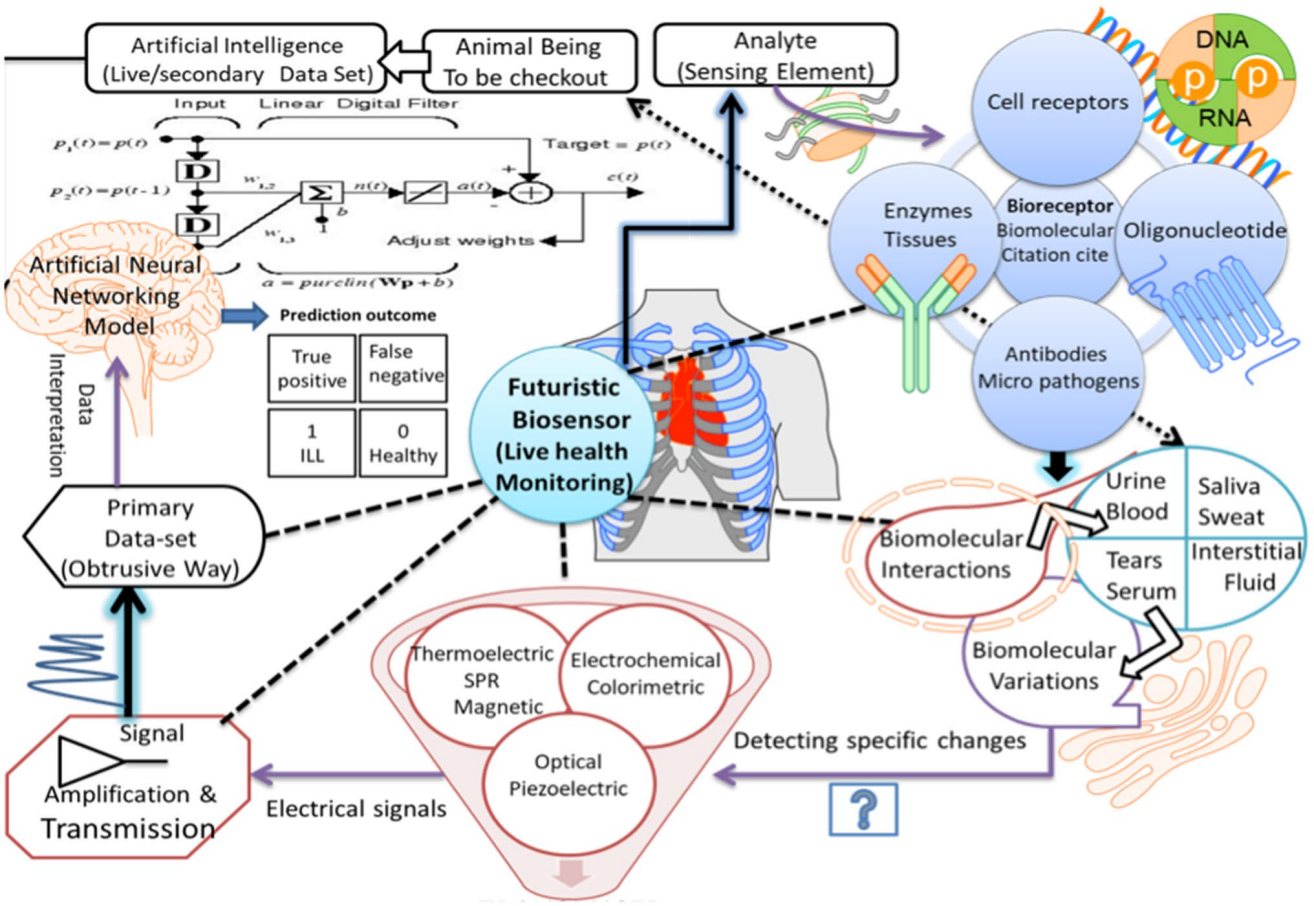
Schematic for holistic diagnosing using different biomarkers, biosensors, and AI-based techniques

Recent advances in the field of artificial intelligence (AI) using machine learning and its successful use in biomedical sciences have cast new areas and tools in creating novel modeling and predictive methods for clinical use including cardiac diseases (Kavakiotis et al. 2017). Cardiac datasets from Keele University, Congenital Heart Disease datasets (CHD) by Government of UK and Cleveland’s heart disease diagnosis data set from the California University in Ervin, etc. have been developed. They store the information that is accessible freely for heart disease prediction using AI. This strategy can be used for the development of point of care (POCT) testing kit that can be used as an important diagnostic tool in a remote area where basic facilities are not available. Furthermore, XPRIZE DeepQ Tricorder biosensor enabled with AI has been developed by Chang et al. (2017) that can accurately diagnose 12 common diseases (anemia, urinary tract infection, diabetes, atrial fibrillation, stroke, sleep apnea, tuberculosis, chronic obstructive pulmonary disease (COPD), pneumonia, otitis media, leukocytosis, and hepatitis A) and capture five real-time vital signs (blood pressure, ECG, body temperature, respiratory rate, and oxygen saturation). The elaboration of biosensors enabled with AI, or next-generation biosensors are probably one of the most promising ways to solve the current problems. Moreover, AI can help in creating more efficient wearable medical devices for real-time monitoring of heart rate, rhythm and thoracic fluid (Pevnick et al. 2017). The holistic viewpoint for the easy and fast CVD diagnostics using different forms of cardiac biomarkers and biosensor is depicted in Fig. 1. Nevertheless, this review will highlight recent advances in wearable devices specifically employed for heart diseases coupled with big data and the internet of things (IoT).

## Characteristics of the ideal smart biosensor

**Fig. 2:**
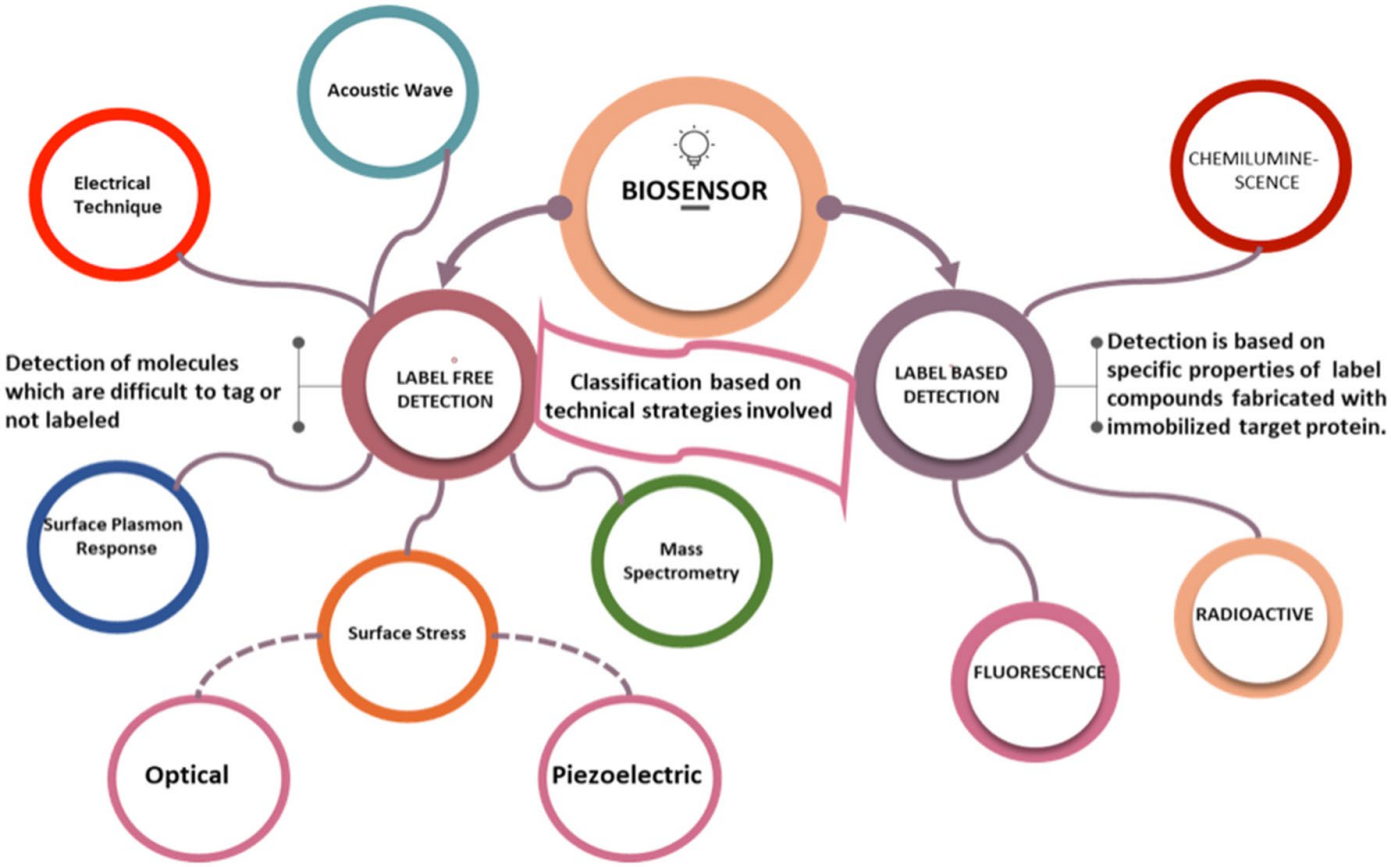
Strategic classification of biosensors on the basis of transduction

**Fig. 3:**
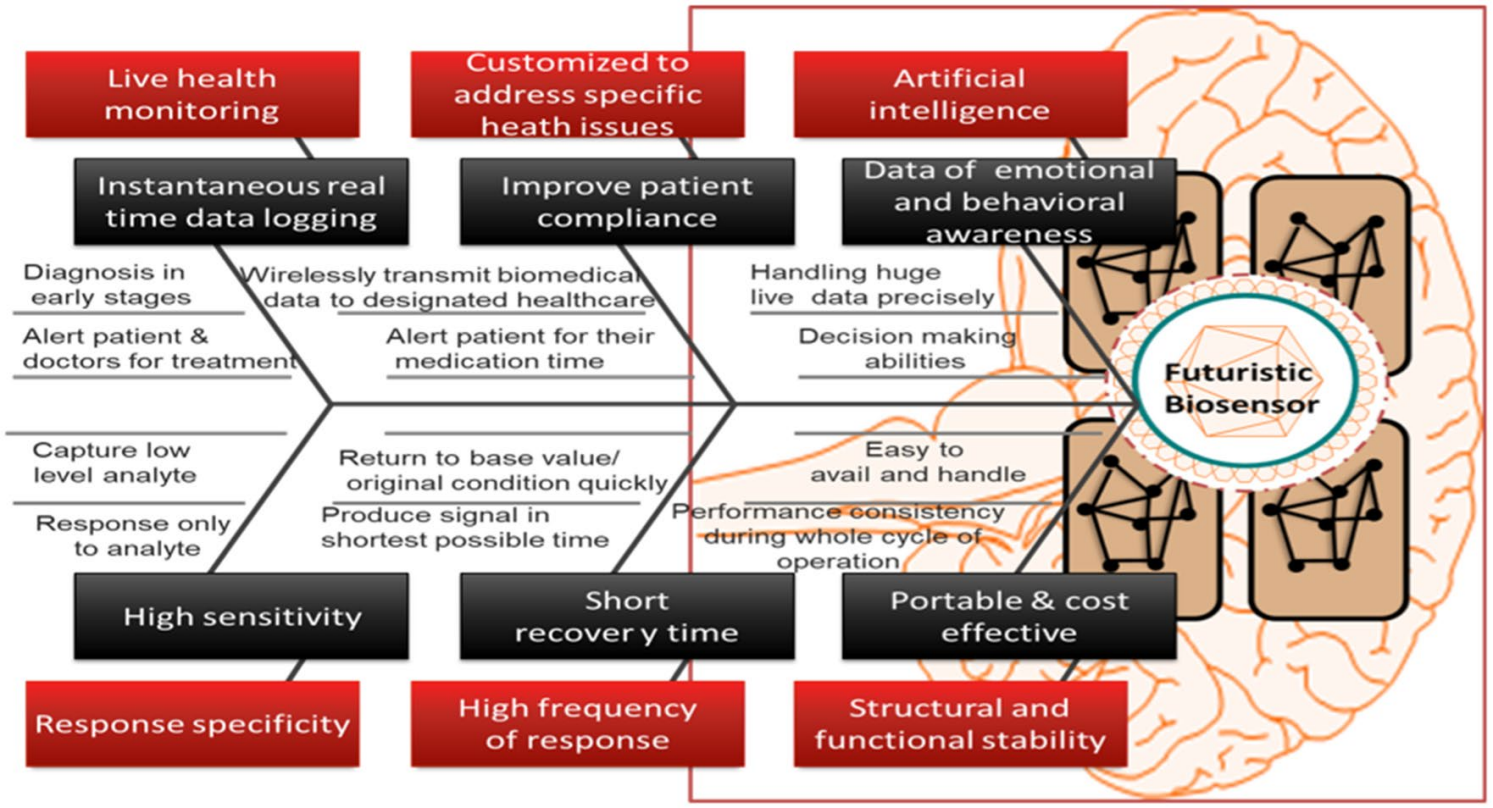
Fishbone diagram representing attributes of an ideal biosensor

An ideal biosensor must promise that it meets the accompanying prerequisites such as response specificity towards the analyte, highly delicate and ready to catch low levels of the analyte (Thévenot et al. 2001). It must have a high recurrence of the reaction and shorter recuperation time. It must have structural and functional stability during its whole cycle of operation and ready to identify little volume analyte. It must be versatile in utility and savvy. It must be customized to address specific health issues and able to transmit the biomedical data wirelessly to the designated healthcare. The design of such biosensors and their ability to generate the huge amount of data for therapeutics provide them real-time decision-making abilities (Stefano and Fernandez 2017). For designing the improved biosensor, the necessary attributes are required. A representative Fig. 2 shows the fishbone diagram representing attributes of an ideal futuristic biosensor. Biosensors having all such properties can respond to many troublesome and uncertain issues. Technical strategies involved in biosensor development are nanotechnologically based, which depends upon either the label-based detection or label-free detection. Also, Nanotechnology enables the manipulation of materials at the nanoscale and has shown potential to enhance sensitivity, selectivity and lower the cost of a diagnosis (Savaliya et al. 2015). Figure 3 shows the strategic classification of biosensors on technical ground based on detection of the pathogen that integrates. Label-based biosensors are highly reliable, and the target detection is based on specific properties of label compounds fabricated with an immobilized target protein, while the label-free biosensors have a wide range of applications in the field of medicine and healthcare because they can detect the molecules which are difficult to tag or not labeled (Citartan et al. 2013; Sang et al. 2016).

Furthermore, biosensors such as fluorescence resonance energy transfer (FRET) microscopy can be used for real-time visualization of second messengers in living cells and detection of alkaline phosphatase in human serum (Kraft and Nikolaev 2017). Also, the G-quadruplex-based fluorometric biosensor is used for detection of histone acetyltransferases (HATs), and histone deacetylases (HDACs) and aptamer-based fluorescence biosensor is used for detection of protein kinase activity to identify biochemical activities associated with various human disease (Wang et al. 2017a, b). However, bio-affinity, microbial, enzyme, and immune-sensors are widely used for diagnostics of many diseases but typically high costs and single time use limit their applications in large sections of the society, especially in developing countries (Hughes et al. 2017). Also, protein engineering approaches have been applied to enhance the therapeutic properties of enzymatic proteins along with modernized purification techniques (Gupta and Shukla 2017; Shukla 2018). Despite this, most of the currently available biosensing systems (such as FRET, surface plasmon resonance (SPR) and electrochemical impedance spectroscopy (EIS), EC, mass spectroscopy, enzyme-linked immunosorbent assay (ELISA), as well as Raman Spectroscopy) suffer from surface saturation due to less accurate target molecule-binding results, sensitivity and limited multiple usage(Wang et al. 2017a, b).

A raised concentration of cholesterol in the blood is one of the major causes for increasing frequency of cardiac arrest and other CVD among human beings, known as hypercholesterolemia (Franco et al. 2011). Also eating and dietary habits of the individuals correspond to the cholesterol abnormality that regulates LDL and HDL (Close et al. 2016). Thus, it is essential to design and develop such biosensors, which can help in the valuation of cholesterol level in blood along with its clinical uses. These biosensors can help for the early assessment of the symptoms in the patients. Due to the easy detection and vitality for therapeutic decision-making, cardiac troponin, which is one of the essential biomarkers for the myocardial inflammations, is being utilized routinely in the set of standard biosensors. Additionally, advancement of biosensors estimating the level of other markers that are non-myocardial tissue specific (such as CRP, copeptin, myeloperoxidase and so forth) can be further useful for the therapeutics (Niotis et al. 2010). These biosensors are said to be ideal in the prevention of CVDs; those can frequently measure the level of CRP, which is the only marker of inflammation that individually forecasts the risk of a heart attack. Moreover, cholesterol oxidase and cholesterol esterase have been utilized as the detecting component for designing a perfect cholesterol biosensor. It is used for the estimation of free and aggregate cholesterol causing a blockage (Arya et al. 2008). Also for the estimation of cholesterol, electrochemical transducers are being efficiently utilized (Zhou et al. 2006). Further different optical transducers, inspecting luminescence, change in color of dye, fluorescence, etc., have likewise been utilized for cholesterol detecting due to the unwavering way of optical transduction (Arya et al. 2008). CRP-based biosensors for simultaneous analyte measurement mainly rely on immuno-sensing technologies with acoustic, optical and electrochemical transducers (Qureshi et al. 2010a). To expand the expository reaction of the cardiac troponin, Silva et al. (2010) intertwined streptavidin polystyrene microspheres to the cathode surface of SPEs. Therefore, ideal biosensor can play a crucial role in the timely and accurate diagnosis of CVD, to spare numerous lives, particularly for the patients enduring the heart attack. Additionally, it can be assumed as a basic part in exact finding, visualization and opportune treatment of the patients through exact and brisk assessment of cardiovascular muscle-particular biomarkers in the blood (Sarangadharan et al. 2018). Accessibility of ideal cholesterol and other biomarker biosensors is turned into a need because of expanding frequencies of CVD and heart failure in contemporary society. Also, a few already have been successfully launched for commercial purposes. To develop an ideal biosensor, some of the parameters should be successfully optimized such as the design of a device, quality issues and enzyme stabilization and effectual decision-making. The cardiac biosensors along with technical strategies involved in their mechanism of working, applications in healthcare, advantages, and limitations are presented in Table 1. To further accelerate diagnosis based on such biosensors for CVD, a superior perception of the bioreagent immobilization and mechanical advances in the microelectronics is required along with the principles of machine learning for data interpretation (Fathil et al. 2017). Nevertheless, for smartphone imaging, the concentration of the analyte was dependent on the color intensity of the electrode. It is carried out using an electrochromic sensor, which detects a highly toxic compound (chlorpyrifos) with a 100 fM and one mM dynamic range, where an electrochromic MIP sensor uses the electrochromic properties of IrOx to detect a certain analyte with high selectivity and sensitivity (Capoferri et al. 2018).

**Table 1:** Recently developed cardiac biosensors along with technical strategies involved in their mechanism of working, applications in healthcare, advantages, and limitations

Biosensor	Technical strategy	Mechanism	Applications	Limitations	Advantages	References
FRET biosensors (tagged biosensor)	Fluorescent-tagged genetically encoded	Förster resonance energy transfer based	Cardiovascular system	Better sensitivity requires an optimal combination of fluorescence nanomaterials	Visualizing cGMP, cAMP, and Ca^2+^ in cells	Thunemann et al. (2014)
Neutrophil gelatinase-associated lipocalin (NGAL) detection biosensor	Measurement based on electrochemical impedance spectroscopy (EIS)	Immobilizing monoclonal antibodies (against NGAL) onto gold disk electrodes	Cardiovascular disease	Detects up to the only certain level of diluted concentration	Point of care biosensor	Gonzalez and La Belle (2012)
Aptamer-based capacitive biosensor	Label-free detection based on non-faradaic impedance spectroscopy	Detection based on charge distribution, of CRP	Cardiovascular disease	Low binding affinity	Reagentless processing	Qureshi et al. (2010a, b)
Single site-specific polyaniline (PANI) nanowire biosensor	Microfluidic channels integrated with nanowire-based biosensors	Detection of the b-type Natriuretic peptide (BNP), myoglobin (Myo), creatine kinase-MB (CK-MB), and cardiac troponin I (cTnI)	Cardiovascular disease (diagnosis of heart failure stages)	Low-cost efficiency	Good specificity with ultra-high sensitivity	Lee et al. (2012)
Substrate-gate coupled FET-based biosensor	Covalent binding immobilization of MAb-cTnI via	Cardiac troponin I (label-free detection)	Cardiovascular disease	Limit of detection	Improved the sensitive detection	Fathil et al. (2017)
Flexible zinc oxide nanostructured biosensor	Detection based on electrochemical impedance spectroscopy and Mott Schottky analysis	Multiplexed and simultaneous detection of two isoforms of troponins	Cardiovascular disease	Limit of detection	Early diagnosis	Shanmugam et al. (2017)
Bienzyme biosensor	Based on functionalized carbon nanotubes (CNTs)	Layer-by-layer amassed and carbon nanotubes/gold nanoparticles-centered	Cardiovascular disease (detection of cholesterol)	The high cost of assembling	High sensitivity, stability, and controllability	Cai et al. (2013)
Nanohybrid composite-based cholesterol biosensors	Doping of engineered g-C_3_N_4_H^+^ nanosheets with cylindrical spongy-shaped polypyrrole	ChOx immobilization on CSPPy-g-C_3_N_4_H^+^nanohybrid composite	Cardiovascular disease (cholesterol detection in human serum)	Poor long-term stability	Cost-effective, biocompatible, eco-friendly	Shrestha et al. (2017)

## Machine learning for biosensor-based device

Data mining methods play a significant role in medical methods. Increasing amount of data and impending cost of computation have allowed machine-learning algorithms to establish its importance in chemical and biosensing applications for clinical and pathological practices (Ching et al. 2018). AI assists clinicians in medical decisions by providing them engraved calculations and offers a promising solution for managing abnormalities. To achieve so, there exist several databases which are developed to portray heart disease classification (Rani 2011). It allows investigation of machine-learning algorithms to deduce conclusive remarks. Among the commonly used databases includes the Long-Term ST Database that stores the ECG recordings of the patients. Apart from that, UCI Repository of Machine Learning Database includes non-invasive and clinical reports of the patients. Also, IQRAA Hospital, Calicut, Kerala, India, includes ECG recordings of the patients between age 40 and 70, and multiparameter Intelligent Monitoring Intensive Care (MIMIC-II) includes physiological parameters and clinical reports of the patient suffering from coronary artery diseases. Moreover, the hierarchical protocol to deduce the results from these databases to diagnose the disease consists of four methods, where the first process is preprocessing, second is feature extraction, third is feature selection, and fourth is learning method (Azuaje et al. 2009).

Preprocessing of data involves removal of noise and the outliers that can be troublesome during analysis for therapeutics. For noise reduction, a low-pass filter and a high-pass filter with a cutoff frequency for removing 20 Hz noise and 0.3 Hz noise, respectively, can be employed, whereas band-rejection can be used to remove noises using a 50-Hz notch filter and power source interference filter (Dolatabadi et al. 2017). Also notch filter and Pan–Tompkins methods are used to eliminate 50-Hz cutoff frequency and identify R-peaks separately (Pan and Tompkins 1985). Another two-way process that is used to analyze the presence of noise in the signal consists of a dynamic time warping technique for segmentation, followed by the Hampel filter to remove the noise from the signal. Moreover, the feature extraction is the process of revealing essential features from the datasets from the various variables (Chan et al. 2004).

Data analytics algorithms for learning method can be segregated as supervised, unsupervised and reinforced. In supervised learning, the data sets are labeled, and the algorithm learns to predict the output from the trained input (Brownlee 2016). Various techniques used for supervised learning are KNN, linear discriminant analysis, support vector machines (SVM), random forest, neural networks (NN) and deep learning (Paiva et al. 2018). The SVM is a statistical learning technique in which the highly nonlinear network is dealt. It expedites to classify random patterns from the dataset, and it is based on structural risk minimization. Hence, it depicts more generalization than that of other learning systems. Also for different applications in medical research, SVM is the most commonly used classifier as it can classify the input samples (Shen et al. 2016). However, training error is minimized from 0.31 to 0.05% while training the dataset using SVM to classify the parameters of CAD detection. For CAD, KNN is one of the most popular classifiers in the machine-learning field. As it does not use any assumptions on the data distribution, therefore, it is also referred to as non-parametric technique. Automatic classification of coronary artery disease (CAD) is achieved using KNN. Also, it has shown that the KNN classifier works superior to SVM classifier for heart irregularity recognition using ECG data. Furthermore, in the fields of medical research, NN is another influential classifier that is widely used because of its easy implementation. NN is based on the structure and functions of biological neural networks. The algorithm processes a solution in the similar way that the human brain works. In the prediction of cardiac abnormalities, NN is successfully implemented as a classifier. Based on it, an effective heart disease prediction system (EHDPS) is developed using a multilayer perceptron neural network with back propagation for predicting the risk level of heart disease and the likelihood of patients getting heart disease. This system uses 15 medical parameters such as age, sex, blood pressure, cholesterol, and obesity for prediction (Singh et al. 2018).

Another non-parametric classifiers used for supervised learning technique is the decision tree (Wah et al. 2018). It is used for classification, regression, and prediction based on the value of a target variable by learning simple decision rules. However, for big data analysis, the random forest is another classifier that is frequently used.

For unsupervised learning the data set is unlabeled, and the algorithm learns to predict the structure or pattern from the input data; clustering and association rule mining are the two most used unsupervised algorithms (Das et al. 2018). While reinforced learning allows the machine software to automatically determine the ideal performance within a specific context, to maximize the result. Therefore, these techniques featuring various machine-learning methods such as deep learning, SVM, Bayesian networks, logistic regression, ensemble methods, NN and the random forest have proved their significance of AI in early cardiac diagnosis. Moreover, to assist physicians in measuring significant clinical parameters, there is a growing inclination towards the use of graphical representations of the patient-specific clinical data and outputs from biosensors (Guidi et al. 2014). Also, to extend the limit of a medical practitioner to the point of the far away area of care diagnosis is even more effective and effectual when it is further coupled with the machine-learning algorithms for prediction and classification purposes.

## Point of care diagnosis using biosensors

For diagnostic purposes, the point of care (POC) can be briefed as a fast, cheap and effective process, which is carried out near the patient ambiance. Integration of biosensors with the wireless capabilities through Bluetooth, Wi-Fi, and GPS has eased the closeness of professional health expert and the home patient (Catherwood et al. 2018). The sensor is coupled with the readout circuit and amplification channels along with the microcontroller to sense and generate the information from the far source. Consumption of power is the limitation in such devices, and self-powered devices are generally designed as so if once a device is implanted, it is impractical to charge the implanted device (Bedin et al. 2017). The objective of POC diagnostics is to rapidly initiate the medication or prognostic where laboratory facilities are less or not available. In the developing and underdeveloped countries, facilities are very less located over the per unit individuals. Therefore, POC diagnostics having biosensors as the nucleus is proving to be a significant protocol, along with the advancements in digitalization (Pandey et al. 2018). Furthermore, development of carbon nanotubes, graphene-metal nanoparticles, has improved the selectivity of POC diagnostics tool (Zhou et al. 2018). Programmable bio-nanochip (p-BNC) system is another biosensor platform with the capacity of learning. It is a “platform to digitize biology” in which sample produces an immunofluorescent signal on agarose bead sensors corresponding to small quantities of patient’s sample, which is further optically extracted and altered to antigen concentrations. The essential components for p-BNC are microfluidic cartridges, automated data analysis software, a portable analyzer, and inbuilt mobile health interfaces (Gaikwad and Banerjee 2018). Additionally to incorporate liquid conveyance, optical recognition, image investigation, and user interface, a compact analyzer instrument was composed speaking to a general framework for gaining, preparing, and overseeing clinical information (McRae et al. 2016). Moreover, Quesada-González and Merkoçi recently discussed the capabilities of nanomaterial for point of care (POC) diagnostics and explained how these materials could help to strengthen, miniaturize and improve the quality of diagnostic devices (Quesada-González and Merkoçi 2018).

**Fig. 4:**
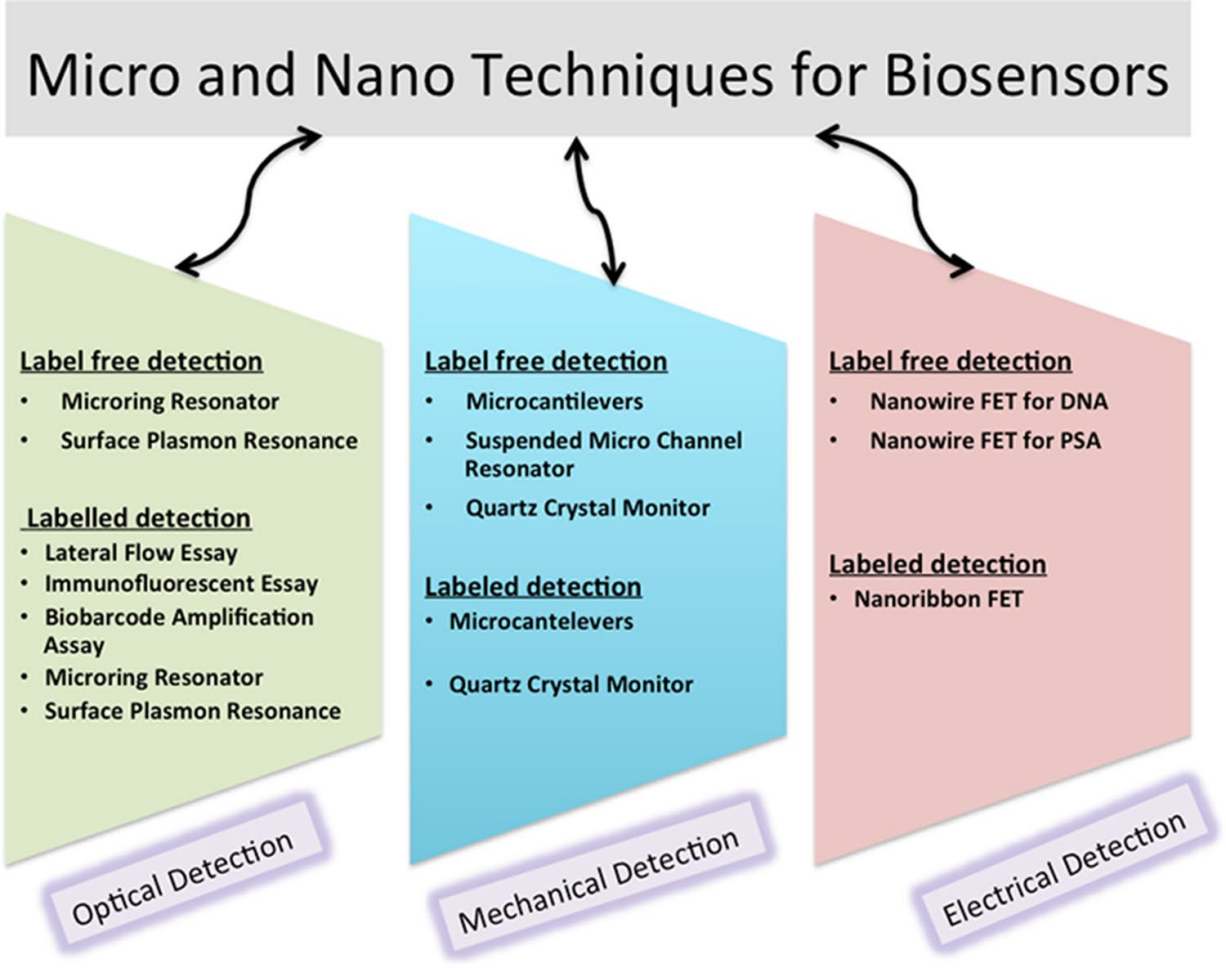
Schematic for label-free and labeled detection in micro- and nanosensing

POC-based applications can be further classified as a lab on a chip, labeled, label-free, nanomaterial-based wearable and wireless (Quesada-González and Merkoçi 2018). Detection mechanisms for the wearables are electrochemical, calorimetric and optical. Conductive ink on the screen-printed electrode on textile and intelligent tattoos and patches are capable of sensing a small number of micro-fluids as biosamples on the epidermis of the skin (Mostafalu et al. 2017). The ‘Lab on a chip’ is the substitute for the complex pathologies and heavy machines in which the biomarker is sensed via micro- and nano-transduction mechanism. These mechanisms include fluorescence intensity measurement, absorbance-based spectrometric, surface plasmon resonance, chemiluminescence, interferometry, amperometric, voltammetric, impedance based, conductometric, thermal, acoustic wave-based detection, paper microfluidic device and lateral flow immunoassay (Siontorou et al. 2017). Lab on chip and microfluidics are robust contenders for delivering the necessary hardware to these electrochemical agents and biosensors. Microfluidic system built by polydimethylsiloxane via soft lithography has various limitations such as cost ineffectiveness and limited accessibility with the introduction of paper-based 3D wax printing technologies such as multi-jet modeling-assisted lab on a chip has gained so much attraction in a very less span of time. Further techniques for optical, mechanical and electrical modes of biosensing under label-free and labeled detection for micro- and nanosensing are shown in Fig. 4. For quantitative detection of CRP, a microfluidic-based system is established by the implementation of a chemiluminescence immunoassay (Hu et al. 2017). This microfluidic-based LOC platform with features of portability, quantitation, and automation establishes a significant strategy for POC diagnosis.

Multiplexed point of care testing (xPOCT) is the simultaneous testing of the various analyte for diseases from a single specimen (Zhang et al. 2018; Dincer et al. 2017). Multiplexing capabilities for POC testing can be grouped as a paper-based system, array-based system, bead-based system and microfluidic multiplexed system with detection techniques lying between optical and lateral flow. Development of user interface devices such as smartphones and smartwatches with such technologies has also opened up the future space for xPOCT (Shanmugam et al. 2017). Sensible cardiovascular observing requires exact heart condition recognition from a cell phone, and wearable-separated photoplethysmogram (PPG) signals through precise recognizable proof and evacuation of commotion. Nearness of commotion especially because of movement ancient rarities emphatically impacts the result of the investigation; consequently denoising of PPG flag yields noteworthy execution viability change while performing Coronary Artery Disease (CAD) identification. Along with this for POC cardiac diagnosis, cardiac scorecard uses a lasso logistic regression approach that converts biomarker data and risk factors into a single score with diagnosable essential information as logistic regression coefficients, which is intended to provide personalized cardiac health assessment (McRae et al. 2016).

## Cardiac big data repositories, IOT, and diagnostics

The era of Internet leads to the connection between people at an exceptional rate; next uprising in this context involves the connectedness of objects: communications, integrating electronics, transducers computing, to create a smart environment through the IoT (Henze et al. 2016). IoT constitutes wearable biosensors along with telemedicine for the preventive health activities and remote medical help along with the continuous monitoring of the patients for chronic cardiac ailments. The above interconnectedness has resulted in big data, which refer to extremely large datasets that cannot be analyzed or interpreted using traditional data processing techniques; therefore to counteract such problem machine-learning algorithms have been evolved to classify and interpret the result (Sun and Reddy 2013). Another definition of big data incorporates union of three terms together, i.e., volume, variety, and velocity, where volume is the quantity of data in a dataset. Thus big data are the quick variation in the large volume of data (Rumsfeld et al. 2016).

To train the machine-learning algorithms with high efficiency and to generate any early interpretation for diagnosis, big data repositories are essential. The source for big data solicitations in cardiovascular medicine includes: administrative databases from pharmaceuticals services, reported data from health survey, data derived from the Internet, medical imaging data, data from all the spectrum of ‘omics’ data, clinical registries and electronic health record data derived from wearable biosensor device. Also, these different data centers can be found at NIH’s BD2K Initiative, CALIBER, CANHEART, Optum Labs, and PCORNet, which include both clinical and patient-driven research networks (Scruggs et al. 2015). Development of analytical platforms due to advances in computational capacity and computer science can accommodate, link, and analyze large, diverse datasets. One such example is Apache Hadoop (Belcastro et al. 2018). Big data imply the use of data science methods, such as data mining or machine learning. Some of the commonly used methods include Bayesian networks, decision-tree learning, cluster analyses, graph analytics, language processing and other data visualization approaches (Johnson et al. 2016). These approaches identify similar patient clusters, creating multiple phenotypes within each disease entity. Also, the hallmark of big data is to combine disparate data sources for predictive analytics, phenomapping, precision health monitoring, clinical decision support and predictive drug management. However, despite the above pros there also exist some cons in big data for cardiology that requires proof such as for complex patients with worse symptoms there exists only a limited amount of findings. Accuracy and reproducibility of precision medicine and drug management are below the satisfaction level, and modeling approaches use assumptions that create skepticism about the validity (Shah and Rumsfeld 2017).

Another IoT-based application incorporating biosensor for cardiac care is a virtual assistant, which is the practice in which the human or nursing staff is replaced by the technology. Biosensors are used for detecting the pathogenic activity in the human body or any kind of abnormality such as hypertension, diabetes, and irregular bowel syndrome that then were processed with the help of data analytics tools to process and formulate the result. Sensely is one such device that is working as VA. It is software as a service (saas) based device being used for regular check up of patients with chronic disorders. It includes biosensors, machine-learning traits and telemedicine that connects the patients automatically to its clinicians upon noticing the threshold symptoms of disorder (Abbott and Shaw 2016). In this way, biosensors embedded with the machine-learning approach solve the purpose of remote care and personalized medicine for effective diagnostics (Vashistha et al. 2018).

## Conclusion

The most significant machine-learning algorithms for the futuristic biosensors and various issues regarding integration of biosensors with the wireless capabilities through Bluetooth, Wi-Fi, and GPS for POC have been investigated. It has been concluded that cardiac big data repositories and internet of things (IoT)-based application integrated with AI along with biosensors for cardiac care can act as a virtual assistant. Real-time monitoring of a patient specific makes the diagnosis of the patient easier and well in time. It is understood that composing the result interpretation via machine learning and data analysis approaches are quite efficient and supports clinical decision-making (Wu et al. 2017). Such parameters are key components for composing size effective and composite smartphone-based devices. The objective of POC diagnostics is to rapidly initiate the medication or prognostic where laboratory facilities are less or not available. Internet of things reduces or eliminates the active human intervention in remote and less facilitated places (Satija et al. 2017). Also, shortly it is plausible that hematocrit, oxygen saturation, HbA1C, lipids, infection, and inflammation biomarkers, which are signs of volume overload or dehydration, can also be integrated into AI technology. Apart from this, touchless or pseudo-touch-based biosensor, which is used to diagnose the disease with the reading of physiological activities operating deep mind algorithms, is the new plot in this field. These days, scientists are quite interested and engaged in developing of the novel, smart and advanced devices such that more specific, sensitive and stable biosensors for theranostics purposes can be invented (Bandodkar et al. 2016). Integrated artificial intelligence tools combining mechanics, biology, chemistry, engineering, etc. is a demand for the present scenario to combat the typical diseases and environmental issues. Thus, AI along with biosensor and bioengineering principles can be considered as a great opportunity to inhibit medical malpractice upon overcoming its limitations of proof and high efficiency shortly.
